# Local abnormal white matter microstructure in the spinothalamic tract in people with chronic neck and shoulder pain

**DOI:** 10.3389/fnins.2024.1485045

**Published:** 2025-01-06

**Authors:** Zhiqiang Qiu, Tianci Liu, Chengxi Zeng, Maojiang Yang, Xiaoxue Xu

**Affiliations:** ^1^Department of Radiology, Affiliated Hospital of North Sichuan Medical College, Nanchong, China; ^2^Department of Pain, Affiliated Hospital of North Sichuan Medical College, Nanchong, China

**Keywords:** chronic neck and shoulder pain, spinothalamic tract, white matter microstructure, DTI, pain ascending pathway

## Abstract

**Objective:**

To investigate differences in the microstructure of the spinothalamic tract (STT) white matter in people with chronic neck and shoulder pain (CNSP) using diffusion tensor imaging, and to assess its correlation with pain intensity and duration of the pain.

**Materials and methods:**

A 3.0T MRI scanner was used to perform diffusion tensor imaging scans on 31 people with CNSP and 24 healthy controls (HCs), employing the Automatic Fiber Segmentation and Quantification (AFQ) method to extract the STT and quantitatively analyze the fractional anisotropy (FA) and mean diffusivity (MD), reflecting the microstructural integrity of nerve fibers. Correlations of these differences with duration of pain and visual analog scale (VAS) scores were analyzed.

**Results:**

No significant differences in the mean FA or MD values of the bilateral STT were observed between people with CNSP and HCs (*p* > 0.05), as indicated by the two-sample t test. Further point-by-point comparison along 100 equidistant nodes within the STT pathway revealed significant reductions in FA values in the left (segments 12–18, 81–89) and right (segments 9–19, 76–80) STT in the CNSP group compared to HCs; significant increases in MD values were observed in the left (segments 1–13, 26–30, 71–91) and right (segments 8–17, 76–91) STT (*p* < 0.05, FWE corrected). Partial correlation analysis indicates that in people with CNSP, the FA values of the STT in regions with damaged white matter structure show a negative correlation with VAS scores and duration of pain, whereas MD values show a positive correlation with VAS scores and duration of pain.

**Conclusion:**

This study found that people with CNSP exhibit white matter microstructural abnormalities in the specific segments of STT. These abnormalities are associated with the patient’s pain intensity and disease duration. The findings offer a new neuroimaging perspective on the pathophysiological basis of chronic pain in the ascending conduction process and its potential role in developing targeted intervention strategies. However, due to the limited sample size and the lack of statistical significance when analyzing the entire spinothalamic tract, these conclusions should be interpreted with caution. Further research with larger cohorts is necessary to validate these results.

## 1 Introduction

Chronic neck and shoulder pain (CNSP) is the most common clinical symptom of cervical spondylosis, defined as persistent pain in the neck and shoulder area lasting more than 3 months (Cohen, 2015). Globally, CNSP exhibits a significant incidence rate, with incomplete statistics indicating an annual prevalence between 10.4 and 21.3% (Hoy et al., 2010). Besides causing long-term pain, CNSP also affects patients’ daily attention, emotions, and cognitive status, greatly impacting personal quality of life (Hofbauer et al., 2001).

The clinical treatment of CNSP mostly involves conservative methods, including medication, exercise, and physical therapy. Some with severe symptoms undergo surgical treatment, but many patients experience long-term pain due to not achieving the desired therapeutic effect, making it the fourth leading cause of disability (Bakhsheshian et al., 2017). Therefore, it is necessary to develop more effective treatment methods. Recently, interventions targeting pain transmission pathways, such as transcranial magnetic stimulation and cranial electrotherapy stimulation, have shown potential in managing chronic pain. These techniques work by modulating neural activity and improving pain processing, helping to reduce pain intensity and enhance quality of life (Shi and Wu, 2023). However, the abnormalities in the brain’s ascending pain pathways in CNSP are still largely unclear.

As we know, nociceptive signals from peripheral sensory organs are transmitted via the spinal cord to the thalamus, where they are modulated and then transmitted to cortical and subcortical structures (Willis and Westlund, 1997). The sensory information of nociceptive (including intensity, location, and nature) is encoded primarily in regions such as the primary somatosensory cortex (S1) and secondary somatosensory cortex (S2) (Coghill et al., 1999). The cognitive modulation of pain processing is believed to be primarily driven by regions within the frontal cortex, such as the anterior cingulate cortex, ventromedial prefrontal cortex, and dorsolateral prefrontal cortex (Lui et al., 2010). Classical brain regions implicated in the affective dimension of pain processing encompass the secondary somatosensory cortex and the anterior insular cortex. These areas are linked to the perception of pain as an unpleasant experience (Schreckenberger et al., 2005). Recent neuroimaging studies have shown that people with CNSP exhibit significant cortical thinning in the S1 region (Woodworth et al., 2019). Additionally, these people also display reduced functional connectivity between the thalamus and the S1 region (Yang et al., 2020). Studies also suggest that in chronic pain states, neural pathways may undergo “central sensitization,” an adaptive change in the central nervous system that leads to the amplification and spread of nociceptive signals, causing an increase in the persistence and intensity of pain (Ji et al., 2018). Based on this, we hypothesize that “central sensitization” induces specific structural and functional alterations in the neural pathways responsible for transmitting nociceptive intensity information. This neural pathway is primarily composed of the spinothalamic tract (STT), which transmits sensory information about nociceptive stimuli (including intensity information) from the lateral columns of the spinal cord to the ventral posterolateral nucleus (VPL) of the thalamus, and then projects it to the S1 and S2 regions for encoding (Wasner et al., 2008).

A previous study (Li et al., 2022) employed tract-based spatial statistics (TBSS) for whole-brain white matter analysis in people with CNSP and found them showed decreased fractional anisotropy (FA) value in the corpus callosum, and increased mean diffusivity (MD) and radial diffusivity values along with decreased FA value in anterior corona radiata compared with healthy controls. However, the TBSS method skeletonizes the fiber tracts, disrupting the correspondence between diffusion parameters and actual anatomical structures, making it difficult to interpret the differences on the skeleton and limiting the analysis of specific fiber tracts (Smith et al., 2006). Therefore, further investigation and exploration are needed to explore the white matter microstructural changes in the STT of people with CNSP. Additionally, the relationship between these white matter microstructural changes and clinical indicators and cognitive function remains unclear.

To test our hypothesis, this study will employ the Automatic Fiber Segmentation and Quantification (AFQ; Yeatman et al., 2012) method to extract the STT from subjects, and quantitatively analyze the FA and MD values that reflect the microstructural characteristics of the neural fibers, exploring the abnormalities in white matter microstructure of the STT in people with CNSP. Furthermore, we collected clinical indicators data from them, including pain intensity, duration of pain, and conducted partial correlation analysis with differences in STT white matter microstructure. This will provide new neuroimaging evidence for evaluating and understanding the pathophysiological basis of such chronic pain in the ascending transmission process, and will also contribute to the development of intervention strategies for the pain ascending pathway.

## 2 Methods

All research procedures were approved by the Ethics Committee of North Sichuan Medical College Affiliated Hospital and conducted in accordance with the Declaration of Helsinki. The approval number is: 2023ER95-1. Written informed consent was obtained from all subjects after they were fully briefed about the study.

### 2.1 Participants

CNSP group: Diagnosed by two experienced pain physicians from the Affiliated Hospital of North Sichuan Medical College according to the chronic pain classification criteria of the International Classification of Diseases 11th Revision (ICD-11; Scholz et al., 2019). Inclusion criteria included: (1) Neck and shoulder pain (with or without radiating arm pain) for at least 3 months, with evidence of cervical degeneration on X-ray or MRI; (2) Aged between 20 and 70 years, right-handed; (3) No MRI contraindications; (4) MRI scans did not reveal spinal compression, and there are no clinical signs of spinal compression; and (5) No significant pain elsewhere in the body. Exclusion criteria included: (1) Significant brain lesions, such as large-area cerebral infarction, cerebral softening, tumors, etc. (Before conducting Imaging acquisition, a routine cranial T2WI scan is performed on the subject. If the subject has Significant brain lesions, they are excluded from this study, and no further Imaging acquisition is conducted.); (2) Primary anxiety disorder, primary depression, Alzheimer’s disease, schizophrenia, or other neurological or psychiatric conditions; (3) Patients unable to undergo prolonged MRI due to severe pain; and (4) Patients with severe underlying cardiac, hepatic, or renal diseases.

HCs group: Inclusion criteria included: (1) Age matched with CNSP group, right-handed; (2) No MRI contraindications; and (3) No acute or chronic pain symptoms at any site. Exclusion criteria included: (1) Significant intracranial lesions; and (2) Neurological or psychiatric conditions.

### 2.2 Clinical indicators assessment

All evaluations were conducted prior to the MRI scans. The average pain intensity of the subjects last week was assessed using a visual analog scale (VAS) going from 0 (zero pain) to 10 (the most intense pain imaginable). Duration of pain was defined as the amount of time between the dates of initial CNSP diagnosis to the date of preoperative brain MRI acquisition.

### 2.3 Imaging acquisition

All MRI scans were performed using a Siemens MAGNETOM Skyra 3.0T MRI scanner and a standard 20-channel head coil. Subjects lay supine on the examination bed, with their heads comfortably positioned and secured with foam to minimize movement, earplugs were worn to reduce external noise, and they were instructed to stay awake, close their eyes, avoid specific thoughts, and keep their heads still.

The T1 high-resolution structural image is acquired at 1 mm isotropic resolution using a three-dimensional (3D) Magnetization Prepared Rapid Gradient Echo (MP-RAGE) sequence with the following parameters: repetition time (TR) = 2,240 ms; inversion time (TI) = 1,130 ms; data matrix = 256 × 256; field of view (FOV) = 256 × 256 mm; slices = 192; slice thickness = 1 mm without gap.

The diffusion magnetic resonance imaging (dMRI) data is acquired at 2 mm isotropic resolution using an echo-planar imaging (EPI) sequence with the following parameters: multiband factor (MB) = 4; 30 noncoplanar diffusion directions with b = 1,000 s/mm^2^; 5 AP (Anterior-to-Posterior) and 5 PA (Posterior-to-Anterior) images with b = 0 s/mm^2^; TR = 10,500 ms; echo time (TE) = 92 ms; data matrix = 128 × 128; FOV = 256 mm × 256 mm; slices = 72; slice thickness = 2 mm without gap.

### 2.4 Data preprocessing

Preprocessing of dMRI data is crucial for subsequent diffusion tensor calculations and tractography, as the quality of preprocessing directly relates to the accurate reconstruction of fiber pathways and the reliable quantitative assessment of neural fiber tracts. Therefore, this study implemented relatively stringent preprocessing steps.

1. Data quality check: Examine the resolution, number of gradient directions, *b*-values, signal-to-noise ratio, artifacts, and head motion in imaging data.
2. Data conversion: Use the dcm2niix tool to convert the collected MRI data from DICOM to NIFTI format.
3. Noise correction: In the case of multi-channel receiver coils, the noise in diffusion data is spatially dependent (Aja-Fernández et al., 2014; André et al., 2014); therefore, we use the Principle Component Analysis of Marchenko–Pastur (MP-PCA; Veraart et al., 2016; Veraart et al., 2016) method for thermal noise correction in MRtrix3 software.
4. Gibbs ringing correction: This artifact is caused by truncation in k-space or finite image sampling (Kellner et al., 2016). Gibbs ringing correction is performed in MRtrix3 software.
5. Echo planar imaging distortions: Diffusion data collection is based on EPI technology, which is susceptible to various distortions caused by magnetic field inhomogeneity. Using b = 0 data with opposite phase encoding directions AP and PA, EPI geometric distortion is corrected using the topup tool in FSL software (Andersson et al., 2003).
6. Motion, eddy current and susceptibility distortion correction: The eddy tool in FSL software is used to correct distortions caused by eddy currents and head motion (Andersson et al., 2016).
7. N4 Bias Field Correction: Diffusion magnetic resonance images exhibit low-frequency intensity shifts, manifesting as intensity inhomogeneities in the image (Banerjee and Maji, 2015). We use the N4BiasFieldCorrection tool in ANTs (Tustison et al., 2010) software for correction.
8. Brain extraction: To enhance the accuracy of spatial registration and limit the analysis scope (fiber tracking) while reducing computational load, the BET (Brain Extraction Tool) in FSL software is used to remove non-brain structures like scalp and skull.
9. Tensor estimation: The DTIFIT tool in FSL software is used to compute the tensor model based on each voxel and calculate the fractional anisotropy (FA) and mean diffusivity (MD) for each voxel.

### 2.5 Automated fiber quantification (AFQ)

**Figure 1:**
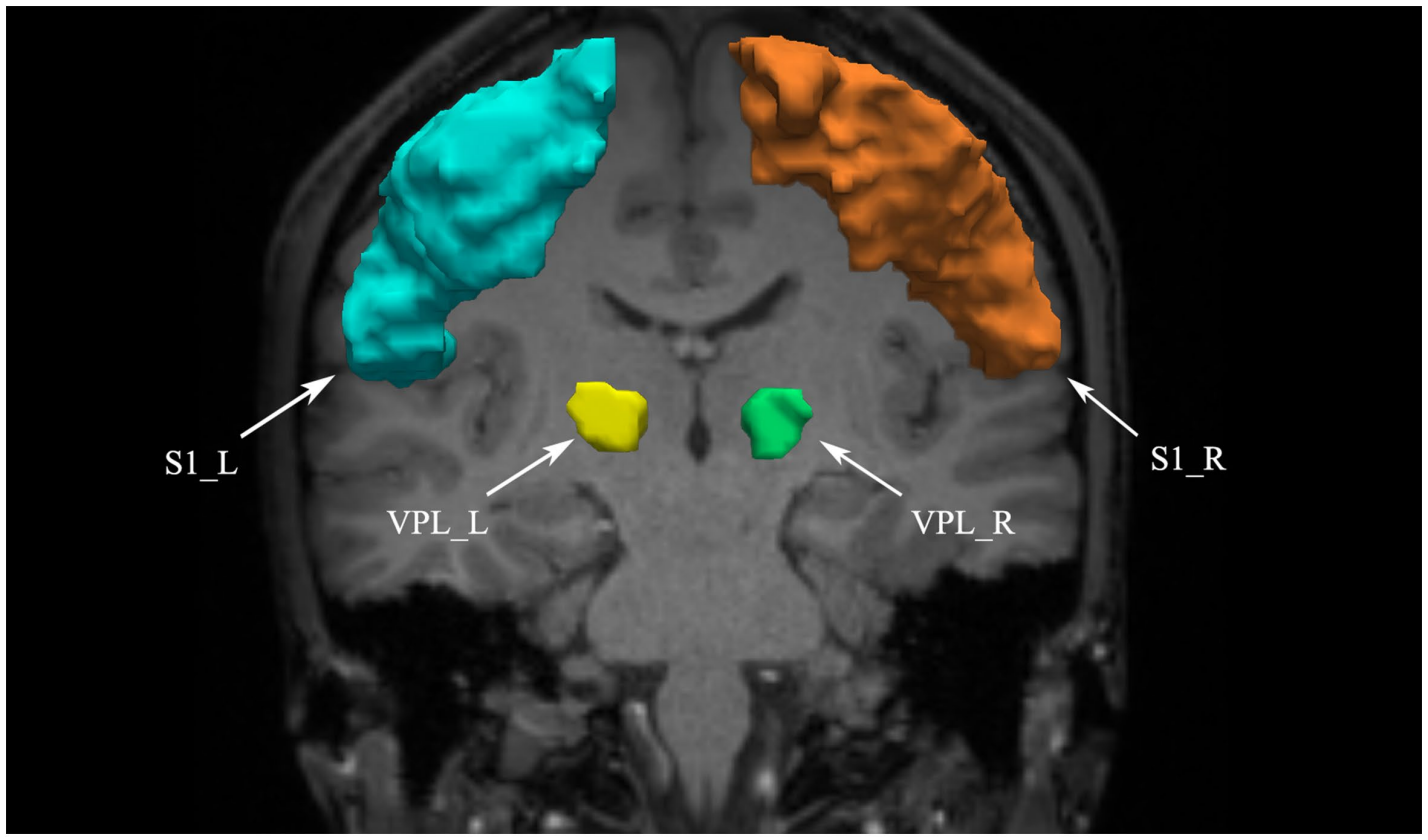
VPL and S1 defined as ROIs. VPL, ventral posterior lateral nucleus of the thalamus; S1, primary somatosensory cortex; L, left; R, right.

1. In the AFQ (Yeatman et al., 2012) software package, manually identify and label the anterior commissure-posterior commissure (AC-PC) line on T1-weighted imaging (T1WI) structural data. This step is mainly used for subsequent registration. AC-PC alignment ensures anatomical consistency between individuals. This improves the reproducibility and accuracy of white matter fiber analysis.
2. Whole-brain tractography: Streamline tracking algorithm is used for whole-brain tractography with parameters set as step length of 1 mm, fractional anisotropy threshold of 0.2, and angle threshold of 30° (Basser et al., 2000).
3. Fiber tract segmentation: Fiber tracts are segmented according to the waypoint ROI procedure described by Wakana et al. (2007). In this procedure, if a fiber passes through two waypoint ROIs that define the trajectory of the fiber tract, it is assigned to a specific fiber group. According to the anatomical definitions by Meola et al. (2016), we define two ROIs in the VPL and S1 areas to extract the STT (see Figure 1). We use ANTs software for nonlinear registration to transform the two ROIs from the standard atlas (Tian et al., 2020; Yeo et al., 2011) to individual brain space.
4. Fiber tract cleaning: After extracting the fiber tracts, an automated cleaning program is applied to remove outlier streamlines from each individual fiber tract. Fibers that are longer than one standard deviation above the average fiber length and deviate spatially more than four standard deviations from the core of the fiber tract are removed (Yeatman et al., 2012).
5. Fiber Tract Quantification: The fiber tract core is determined by resampling each fiber into 100 evenly spaced nodes and calculating the mean location of each node. Diffusion properties, such as fractional anisotropy (FA) and mean diffusivity (MD), are calculated at each node using spline interpolation. At each node, these properties are summarized by taking a weighted average of the diffusion values from all fibers. The weight assigned to each fiber depends on the probability of its membership in the fascicle, which is calculated based on the fiber’s Mahalanobis distance from the fiber tract core (Yeatman et al., 2012).

### 2.6 Statistical analysis

Pearson’s chi-squared test was used to compare gender differences between groups, while two-sample *t* test was used to compare age differences between groups.

For each subject’s bilateral STT, we calculated the average FA and average MD value across the core of the tract and then used two-sample *t* test to compare these values between different groups, considering *p* < 0.05 as statistically significant.

Furthermore, considering that the STT in people with CNSP may be partially damaged, statistical analyses of average FA and average MD values might obscure these local differences. Therefore, we evaluated local differences between groups by comparing FA and MD values at 100 nodes along the STT pathway. To account for multiple comparisons, we used non-parametric permutation test to assess differences between groups, with 5,000 permutations, followed by threshold-free cluster enhancement (TFCE) with the family-wise error (FWE) correction to identify segments where FA and MD values differed significantly between groups (*p* < 0.05) (Banfi et al., 2019).

To investigate the relationship among people with CNSP’s diffusion properties, duration of pain, and the VAS scores, we calculated the average FA and average MD values in significantly different segments, and a partial correlation analysis was performed. FDR correction was performed to account for multiple comparisons, with *p* < 0.05 indicating statistical significance.

## 3 Results

### 3.1 Clinical and demographic characteristics

In this study, 34 people with CNSP were initially included, with 1 discontinuing the MRI scan due to pain and 2 excluded for excessive head motion during data quality assessment, ultimately including 31 people with CNSP and 24 healthy controls. There were no significant statistical differences in age and gender between the two groups (*p* < 0.05). Clinical and demographic results for the CNSP group and HCs group can be found in Table 1.

**Table 1:** Demographic and behavioral data.

	CNSP (*n* = 31)	HCs (*n* = 24)	*p* value
Gender (male/female)	15/16	11/13	0.851
Age (years)	49.68 ± 7.34	47.45 ± 8.52	0.286
Duration of pain (months)	38.56 ± 17.34	–	–
VAS	6.12 ± 1.34	–	–

CNSP, chronic neck and shoulder pain; HCs, healthy controls; VAS, visual analog scale.

### 3.2 Inter-group comparison of average FA and MD values in bilateral spinal thalamic tracts

The average FA values of the bilateral STT in the CNSP group were not significantly different from those in the HCs group (left STT *p* = 0.166, right STT *p* = 0.245, both >0.05). Similarly, the average MD values of the bilateral STT in the CNSP group were not significantly different from those in the HCs group (left STT *p* = 0.149, right STT *p* = 0.218, both >0.05; see Table 2).

**Table 2:** Comparison of average diffusion index of spinal thalamic tract between groups.

Diffusion metrics	CNSP	HCs	*t*	*p*
FA_L	0.532 ± 0.036	0.539 ± 0.043	−1.145	0.166
FA_R	0.541 ± 0.037	0.547 ± 0.046	−0.884	0.245
MD_L	0.779 ± 0.069	0.769 ± 0.062	1.122	0.149
MD_R	0.774 ± 0.063	0.765 ± 0.052	0.915	0.218

CNSP, chronic neck and shoulder pain; HCs, healthy controls; FA, fractional anisotropy; MD, mean diffusivity.

### 3.3 Segmental comparison of diffusion metrics in bilateral spinal thalamic tracts between groups

**Figure 2:**
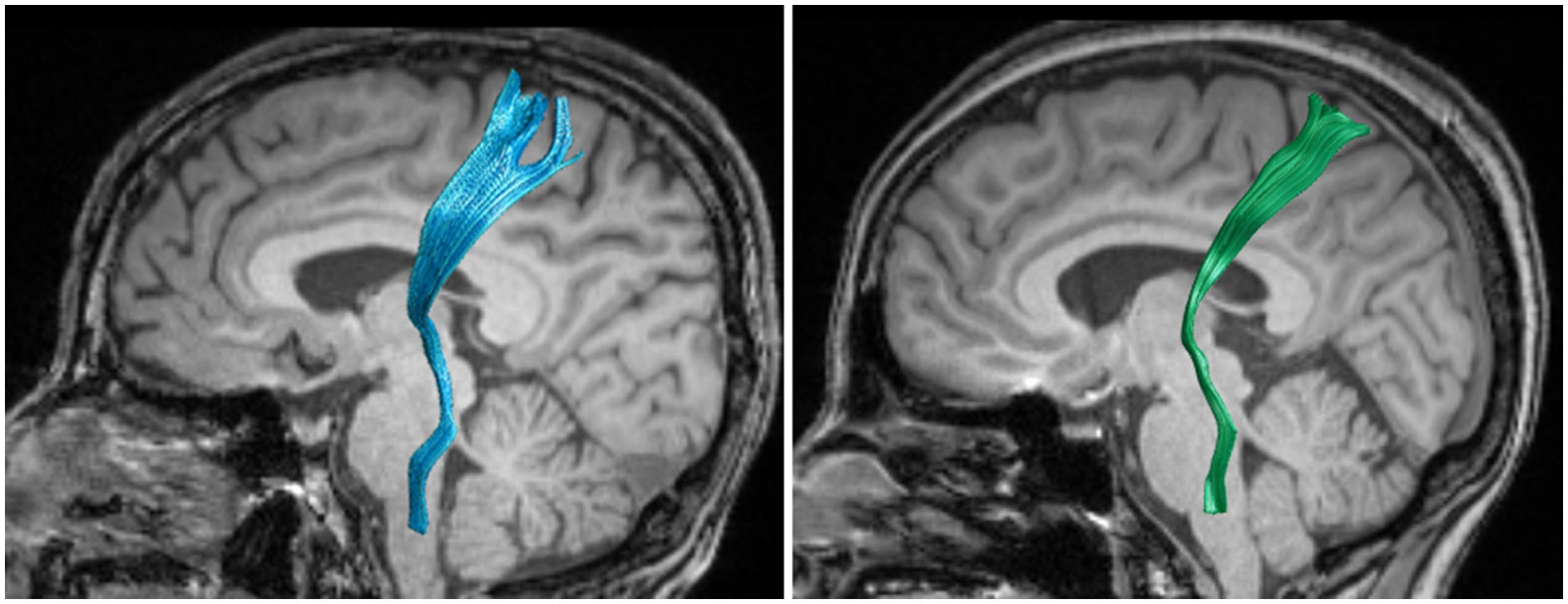
Bilateral spinothalamic tract fiber tracks. The figure demonstrates bilateral spinothalamic tract of a representative people with CNSP, with the left image showing the left spinothalamic tract and the right image showing the right spinothalamic tract.

**Figure 3:**
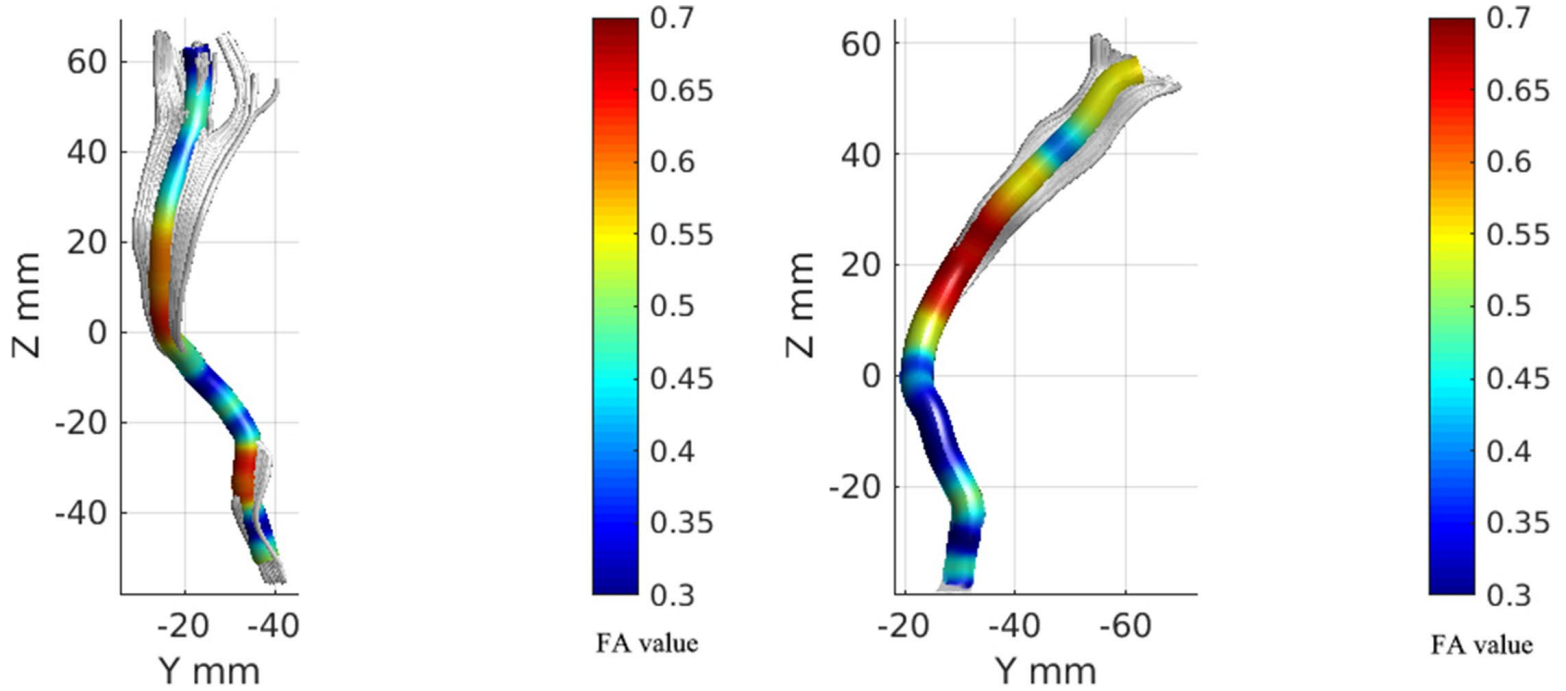
FA value rendering images of bilateral spinal thalamic tracts in one case of people with CNSP. The left image represents the FA value rendering of the left spinothalamic tract, while the right image represents the FA value rendering of the right spinothalamic tract.

Considering that the STT in people with CNSP may only be locally damaged, statistical analysis of average FA and average MD values might obscure these local differences. We further performed node-by-node comparisons of FA and MD values at 100 equidistant nodes along the STT pathway. Figures 2, 3 show the bilateral STT fiber tracing results and FA value renderings along their trajectories for a representative people with CNSP.

**Figure 4:**
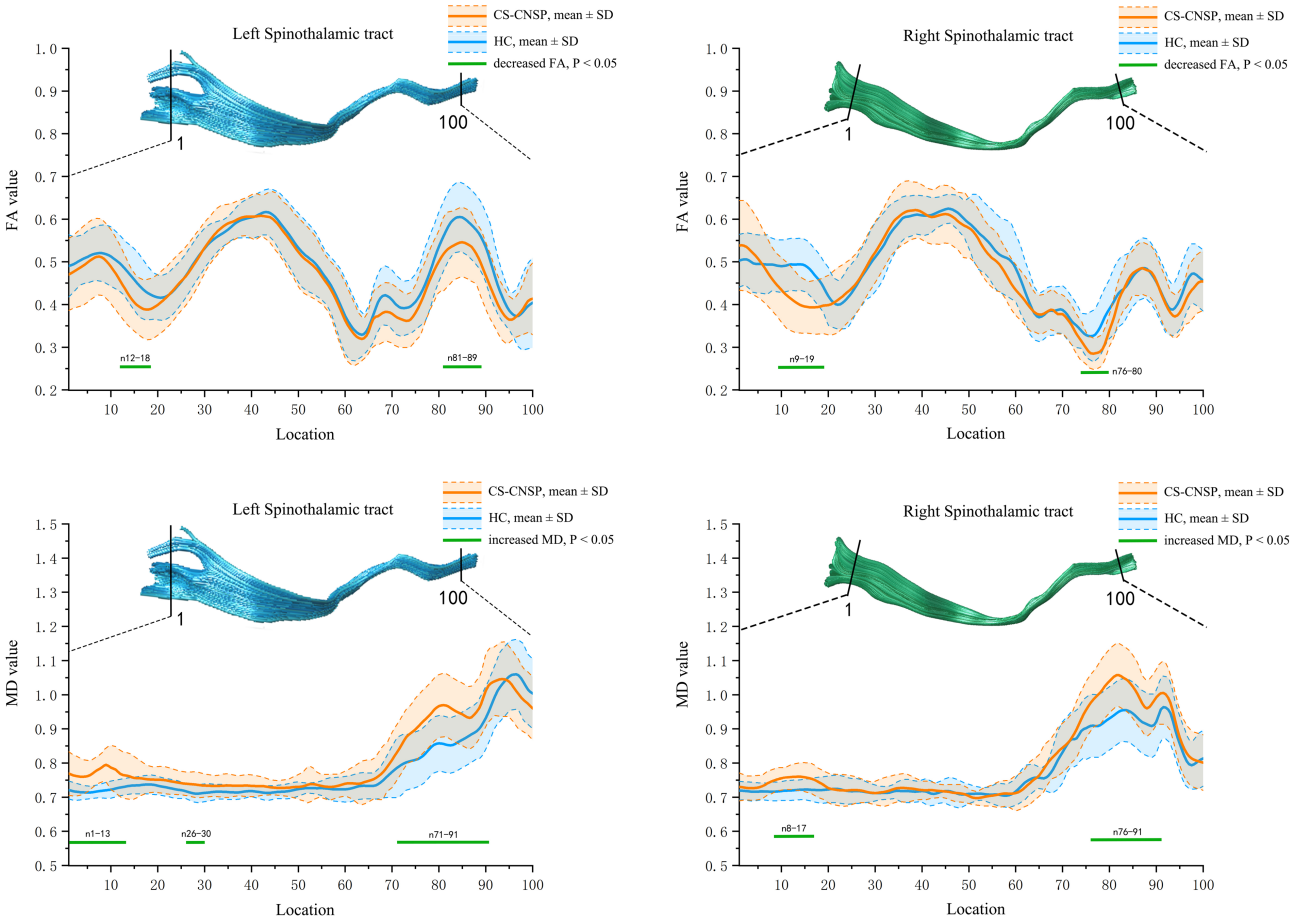
Group comparison of FA and MD profiles. CNSP, chronic neck and shoulder pain; HCs, healthy controls; FA, fractional anisotropy; MD, mean diffusivity; SD, standard deviation.

Figure 4 shows the FA and MD value curves at 100 equidistant nodes along the bilateral STT for CNSP and HC groups, and the green line segment highlighting sections with statistically significant differences (*p* < 0.05, FWE corrected).

FA value comparison: The FA values in the left STT (segments 12–18, 81–89) and right STT (segments 9–19, 76–80) of the CNSP group were significantly lower compared to the HC group (*p* < 0.05, FWE corrected).

MD value comparison: The MD values in the left STT (segments 1–13, 26–30, 71–91) and right STT (segments 8–17, 76–91) of the CNSP group were significantly higher compared to the HC group (*p* < 0.05, FWE corrected).

### 3.4 Partial correlation analysis of bilateral spinal thalamic tract diffusion metrics with clinical indicators

Based on the statistical results of point-by-point comparisons of quantified parameters of white matter fiber tract of the bilateral STT between the CNSP and HCs groups, the following were included in the correlation analysis: FA values of the left STT (segments 12–18), left STT (segments 81–89), right STT (segments 9–19), right STT (segments 76–80), MD values of the left STT (segments 1–13), left STT (segments 26–30), left STT (segments 71–91), right STT (segments 8–17), and right STT (segments 76–91).

**Figure 5:**
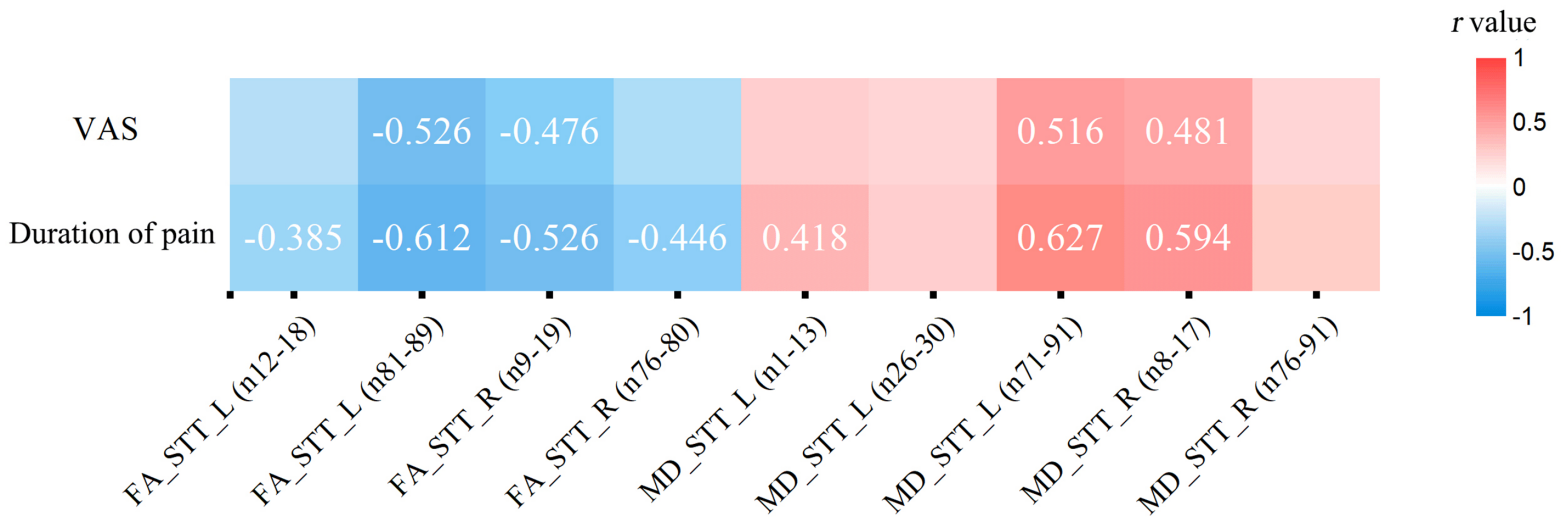
Partial correlation analysis of spinothalamic tract diffusion metrics with clinical measures. Only correlation coefficients (r) with statistical significance (*p* < 0.05, FDR corrected) are marked. VAS, visual analog scale; FA, fractional anisotropy; MD, mean diffusivity; STT, spinothalamic tract; L, left; R, right.

The results indicate that the duration of pain shows a negative correlation with the average FA value of the left STT (segments 12–18) (*r* = −0.385, *p* = 0.038, FDR corrected). VAS scores (*r* = −0.526, *p* = 0.004, FDR corrected) and the duration of pain (*r* = −0.612, *p* < 0.001, FDR corrected) show a negative correlation with the average FA value of the left STT (segments 81–98). VAS scores (*r* = −0.476, *p* = 0.012, FDR corrected) and the duration of pain (*r* = −0.526, *p* = 0.005, FDR corrected) are negatively correlated with the average FA value of the right STT (segments 9–19). The duration of pain (*r* = −0.446, *p* = 0.013, FDR corrected) is negatively correlated with the average FA value of the right STT (segments 76–80). The duration of pain is positively correlated with the average MD value of the left STT (segments 1–13) (*r* = 0.418, *p* = 0.019, FDR corrected). VAS scores (*r* = 0.516, *p* = 0.006, FDR corrected) and the duration of pain (*r* = 0.627, *p* < 0.001, FDR corrected) are positively correlated with the average MD value of the left STT (segments 71–91). VAS scores (*r* = 0.481, *p* = 0.011, FDR corrected) and the duration of pain (*r* = 0.594, *p* = 0.002, FDR corrected) are positively correlated with the average MD value of the right STT (segments 8–17) (see Figure 5). The statistically significant results (*p* < 0.05, FDR corrected) are annotated in the figure with the corresponding correlation coefficients *r*. Among these, the strongest correlations for FA and MD values are with the disease duration in people with CNSP, followed by VAS scores.

## 4 Discussion

In this study, the AFQ method was used to extract STT from people with CNSP and HCs, and quantitatively analyzed FA and MD values that reflect the microstructural characteristics of fiber tracts. It explored differences in the white matter microstructure of the STT in people with CNSP and examined the associations of these microstructural differences in STT with VAS scores and duration of pain. Our results showed segmental differences in the STT white matter microstructure of people with CNSP, which were also related to pain intensity and duration of pain. These findings and their potential interpretations will be discussed in the following text.

DTI technology can non-invasively reconstruct the three-dimensional structure of brain white matter fibers. By quantifying the diffusion capabilities of water molecules along the axial and transverse directions of nerve fibers, DTI provides an effective tool for revealing abnormalities in the microstructure of white matter at the microscopic level (Beaulieu, 2002). This study uses DTI’s two core quantitative parameters, FA and MD, to investigate differences in the microstructure of white matter in the STT of people with CNSP. FA is a metric that measures the anisotropy of water molecule diffusion, reflecting the degree of directional preference for water molecules diffusing along nerve fibers. A reduction in FA values typically suggests impaired directionality and coherence of nerve fibers, implying a decrease in the structural integrity of white matter (Budde et al., 2009). On the other hand, MD measures the average diffusion rate of water molecules within a voxel, and can be seen as an indicator of the complexity of white matter microstructure. In areas where the white matter structure becomes more complex or where there is an increase in crossing and branching of fiber tracts, MD values tend to decrease; conversely, an increase in MD values may reflect demyelination or edema of fiber tracts (Le Bihan et al., 2001).

In this study, we initially compared the average diffusion metrics of bilateral STT between people with CNSP and HCs. The results showed no statistically significant differences in either FA or MD values between the two groups (*p* > 0.05). We speculate this may be related to the averaging of diffusion metrics across the entire fiber bundle, which could potentially obscure the underlying local white matter microstructural damage. Travis et al. (2015) similarly discovered this phenomenon in their research on the cerebellar white matter pathways.

To further investigate whether there is localized white matter microstructural damage in STT in people with CNSP, we compared the FA and MD values at 100 equidistant nodes along the STT pathway. The results indicated that, compared to the HCs group, people with CNSP showed significantly decreased FA values, which may reflect impaired directionality and coherence of nerve fibers, implying a decrease in the structural integrity of white matter (Budde et al., 2009). Additionally, increased MD values were observed, which could indicate extracellular water accumulation or alterations in tissue structure, potentially consistent with demyelination or edema in these fiber tracts (Le Bihan et al., 2001). These findings are similar to those of He et al. (2021), who also identified localized white matter microstructural damage in STT in patients with primary dysmenorrhea using a tractography atlas-based analysis (TABS).

The pathophysiological mechanisms responsible for microstructural alterations in the STT white matter in individuals with CNSP are not fully understood. The VPL of the thalamus plays a pivotal role in this process, acting as the principal relay station for somatic sensory information from the peripheral receptors to the cerebral cortex. The VPL receives afferent signals from primary sensory neurons (excluding the face) via the STT and relays this information to the S1 (Morel et al., 1997). Additionally, the VPL is involved in the initial stages of nociceptive signal processing and modulation (Apkarian et al., 2005). The S1 area, located in the postcentral gyrus, is a key brain region for processing somatic sensory inputs, responsible for receiving and processing sensory information such as nociception, touch, and temperature from various parts of the body. This information is transmitted through the spinal cord and thalamus (such as the VPL nucleus) to the S1 area, where it is identified, localized, and processed, allowing the S1 area to accurately pinpoint the location of nociceptive and assess the intensity of the stimulus (Kaas, 1993). The fibrous pathways between the thalamus and the S1 area form the lateral pain system, which is widely regarded as the basis for normal individuals to detect and perceive the intensity of noxious stimuli (Moulton et al., 2005). Some scholars believe that the continuous stimulation of chronic pain may cause prolonged overactivation of neurons in the pain conduction pathways, which can lead to structural and functional changes in neurons and glial cells, resulting in damage to the microstructure of the white matter (Lin et al., 2017). Other researchers suggest that chronic pain may lead to changes in neural plasticity in the brain, including neuronal reorganization and synaptic remodeling, which may affect the function and structure of the pain conduction pathways, causing damage to the white matter microstructure (May, 2008). Furthermore, the relationship between this pain and brain white matter abnormalities may be bidirectional. On one hand, chronic pain can cause microstructural changes in the STT white matter; on the other hand, damage or dysfunction of neurons in the STT may lead to abnormal amplification of nociceptive signals, causing patients to experience more intense pain (Ji et al., 2014). This indicates that CNSP may not only be caused by stimulation or compression of nerve roots but may also be related to changes in pain-related white matter pathways in the central nervous system.

In this study, damage to the STT in people with CNSP is anatomically concentrated in two key regions: the segments near the pons (segments 81–89, 76–80, 71–91, 76–91) and the white matter segments near the primary somatosensory cortex (segments 12–18, 9–19, 1–13, 8–17). The pons, located in the middle of the brainstem, is a critical structure connecting the brain, cerebellum, and spinal cord, containing numerous ascending and descending conduction fibers responsible for signal integration and transmission (Wang et al., 2022). The densely packed fiber pathways may result in high functional loads. Similarly, the white matter regions near the cortex are characterized by a dense network of neural pathways, making them potentially more susceptible to functional overload (Shinohara et al., 2020). Therefore, we speculate that the spinothalamic tract located in these regions may be more susceptible to repetitive high-frequency stimulation (nociceptive signals induced by chronic pain), leading to a greater susceptibility to damage to the microstructure of white matter.

In our results, the FA values of the STT white matter fibers in people with CNSP showed a negative correlation with VAS scores and duration of pain, while the MD values were positively correlated with these measures. Additionally, this alteration in white matter microstructure was most strongly correlated with the duration of CNSP, followed by VAS scores. This suggests that the impairment of the STT white matter microstructure in people with CNSP is closely related to the prolonged existence of chronic pain, and this damage increases as the pain persists. These fiber pathways may play an important role in discerning the intensity and duration of pain. Some scholars believe that prolonged pain stimulation may lead to plastic changes in the central nervous system, namely central sensitization, involving neurons and their connections in the conduction pathway. The microstructural changes in the fiber tracts may reflect the degree of this central sensitization, and thus positively correlate with the patient’s disease progression and pain intensity (Woolf and Salter, 2000). Some researchers also believe that damage to the white matter microstructure in the conduction pathways may affect the conduction speed of nerve impulses, leading to delayed or disrupted pain signal transmission. This change in conduction speed may be related to the intensity and duration of pain experienced by the patient (Fields, 2004).

## 5 Limitations

(1) This study, a cross-sectional analysis, revealed abnormalities in the STT of people with CNSP. However, it is still unknown whether these abnormalities impact the effectiveness of pain treatments or whether the damaged white matter microstructure will recover after pain relief, necessitating further longitudinal research exploration. (2) Some people in this study exhibited lateralized pain symptoms (pain only on one side of the neck and shoulder), but it remains unclear if this lateralized pain leads to lateralized changes in conduction pathways; future research could focus on this by increasing the sample size and conducting subgroup analyses on lateralized pain. (3) This study utilized DTI technology for visualizing fiber tracts, however, this technique cannot depict crossing or branching fibers (Tuch, 2004). Future imaging could employ high-angular resolution diffusion imaging (HARDI; Ozarslan and Mareci, 2003) with higher b-values and more diffusion gradient directions, or diffusion spectrum imaging (DSI; Wedeen et al., 2008) based on the probability density function with multiple *b*-values and directions. These imaging models overcome DTI’s limitations, capable of displaying more complex structures such as crossing and branching fibers, providing more accurate and comprehensive information about fiber trajectories and connections. (4) We acknowledge that while there were no significant differences in age between the two groups, the wide age range may have introduced variability in the results due to age-related microstructural changes in the brain (Salami et al., 2012). Additionally, the wide range in pain duration among participants could also influence the outcomes. Future studies should aim to recruit sufficient participants to enable subgroup analyses and adequately account for these sources of variability.

## 6 Conclusion

This study found that people with CNSP exhibit white matter microstructural abnormalities in the specific segments of STT. These abnormalities are associated with the patient’s pain intensity and disease duration. The findings offer a new neuroimaging perspective on the pathophysiological basis of chronic pain in the ascending conduction process and its potential role in developing targeted intervention strategies. However, due to the limited sample size and the lack of statistical significance when analyzing the entire spinothalamic tract, these conclusions should be interpreted with caution. Further research with larger cohorts is necessary to validate these results.

## Data Availability

The raw data supporting the conclusions of this article will be made available by the authors, without undue reservation.
